# REIMAGINE: A central nervous system basket trial showing safety and efficacy of vafidemstat on aggression in different psychiatric disorders

**DOI:** 10.1111/pcn.13800

**Published:** 2025-02-12

**Authors:** Marc Ferrer, Vanesa Richarte, Laura Gisbert, Jordi Xaus, Sonia Gutierrez, Maria Isabel Arevalo, Michael Ropacki, Roger Bullock, Carlos Buesa, Josep Antoni Ramos‐Quiroga

**Affiliations:** ^1^ Department of Psychiatry Hospital Universitari Vall d'Hebron Barcelona Spain; ^2^ Oryzon Genomics S.A. Cornellà de Llobregat Barcelona Spain

**Keywords:** aggression, attention‐deficit/hyperactivity disorder, autistic spectrum disorder, borderline personality disorder, vafidemstat

## Abstract

**Aim:**

Vafidemstat is a brain‐penetrant, orally bioavailable, small molecule irreversible inhibitor of the histone lysine‐specific demethylase KDM1A (also known as LSD1), which corrects memory deficits and behavior alterations including aggression and social interaction deficits in preclinical models.

**Methods:**

Here, we report the results of REIMAGINE, a phase IIa, single‐center, open‐label, one‐arm basket trial that evaluated the safety and efficacy of vafidemstat on aggression in adult patients with borderline personality disorder (BPD), attention‐deficit/hyperactivity disorder (ADHD), and autistic spectrum disorder (ASD). Participants received 1.2 mg/day of vafidemstat for 8 weeks.

**Results:**

Vafidemstat was shown to be safe and well tolerated, and no drug‐related clinically significant adverse events were observed. Furthermore, all neuropsychiatric scales assessed showed notable efficacy signals, whether assessing agitation/aggression (Clinical Global Impression for Severity [CGI‐S] and Clinical Global Impression for Improvement [CGI‐I] and Neuropsychiatric Inventory [NPI] questionnaire for Agitation‐Aggression [NPI‐AA]), overall patient functioning (total NPI), or disease‐specific features (Attention‐Deficit/Hyperactivity Disorder Rating Scale [ADHD‐RS] and Borderline Personality Disorder Checklist [BPDCL]). Statistically significant improvements were observed in the aggregated data (all participants) and for each of the three disease groups independently. Changes were evident within the first 2 weeks of treatment.

**Conclusion:**

In summary, the REIMAGINE study supports that vafidemstat is safe, well tolerated, and causes a significant and consistent reduction in agitation/aggression and nonaggression features in BPD, ADHD, and ASD. These data support continuing the development of vafidemstat as a new treatment option for these psychiatric disorders.

Basket trials are commonly used in oncology to assess the efficacy of a new therapy on multiple cancer types that share common molecular alterations or risk factors.^1^ This type of trial facilitates the study of a single compound across different indications, usually in an open‐label adaptive design, to provide an initial proof of concept on the efficacy of the new therapy, thus guiding future definitive studies. Such studies have not been a focus of central nervous system (CNS) research, mainly because CNS disorders have traditionally been deemed distinct and therefore researched separately. However, as in oncology, gene transcription disequilibria are characteristic of many neurodegenerative and psychiatric disorders.^2^, ^3^, ^4^, ^5^, ^6^

KDM1A, a histone 3 lysine 4 demethylase that forms part of transcription regulation complexes, plays a key role in the epigenetic regulation of CNS during development and, after birth, in neurite morphogenesis. KDM1A has been reported as the most abundant histone demethylase in the prefrontal cortex,^7^ which is key in the control of aggressive behavior, where it has been implicated in the control of immediate early genes (IEGs) transcription.^8^

Vafidemstat (ORY‐2001) is a highly brain‐penetrant, orally bioavailable, small molecule covalent inhibitor of KDM1A. In preclinical models, vafidemstat corrects memory deficits and behavior alterations, including aggression and social interaction deficits in senescence‐accelerated prone 8 (SAMP8) mice and social avoidance in the rat‐rearing isolation model, while not acting as a sedative or anxiolytic drug.^8^ In addition, vafidemstat corrects the abnormal response to stress of IEGs in the prefrontal cortex, induces genes required for cognitive function, and reduces neuroinflammation in the brain of mouse models of CNS disease.^8^, ^9^ Interestingly, many key genes modulated by vafidemstat are differentially expressed in the brain of patients with Alzheimer disease and other diseases of the CNS.^8^ These data provide support for evaluating vafidemstat as a nonsedative treatment for behavior disturbances, particularly agitation/aggression (A/A), associated with a variety of psychiatric and neurodegenerative diseases.

Borderline personality disorder (BPD) is a common mental health disorder with an estimated prevalence in the general adult population between 0.5% and 5.9%^10^, ^11^ and higher in clinical settings. For instance, prevalence in primary care clinics is 6% versus 10% of psychiatric outpatients and 20% in psychiatric inpatient units.^12^, ^13^ As compared with depression, patients with BPD have a deeper functional impairment and higher use of medical, mental health, and social services.^14^

A/A is a dysfunctional behavior present in many patients with neurological and psychiatric disorders, significantly impacting patients' functioning and quality of life. As in many other psychiatric disorders, A/A is also often present in patients with BPD, worsening patients' already unstable interpersonal relationships. In BPD, aggression often includes self‐damaging impulsivity, recurrent suicidal behaviors, threats, and self‐injurious behavior, as well as inappropriate, intense anger or difficulty controlling anger. Altogether, internal, and external A/A, impulsivity, and emotional instability result in severe and significant functional impairment and disability, as well as a lower quality of life for patients with BPD.

Patients with BPD frequently exhibit internalized self‐harm aggressive behaviors including suicidal thoughts, gestures, and/or attempts, which may occur several years after the first presentation of symptoms. Suicide is common in people with borderline personality disorder and may occur several years after the first presentation of symptoms.^15^ The rate of completed suicide in people with BPD has been estimated to be ≈10%, 50‐fold higher than in the general population.^16^ Despite the high burden associated with BPD, no pharmacological therapy has been approved for BPD.

Attention‐deficit/hyperactivity disorder (ADHD) has a prevalence of 2.5% to 4% in the adult population.^17^ Core features include difficulty in paying attention, hyperactivity, and impulsivity. Patients with ADHD also exhibit A/A behavior, which can affect up to 50% of patients.^18^ This can lead to unstable relationships, poor work performance, and low self‐esteem. Long‐acting stimulants are the most prescribed medications for this condition in adults, but ≈30% of the patients do not respond to this pharmacological treatment.^19^

Autistic spectrum disorder (ASD) shows early and persistent behavioral changes, with A/A toward caregivers occurring in 69% of cases and toward strangers in 49% of cases.^20^ A prevalence rate of ≈1% has been described in adults.^21^ Currently, risperidone and aripiprazole are the only approved drugs to specifically treat aggression in ASD, but they are associated with significant side effects, such as sedation, weight gain, tardive dyskinesia, and increased prolactin levels.^22^

Together, these three conditions present a significant unmet need, with limited therapeutic options, and all of them with frequent episodes of A/A. Antipsychotics,^23^, ^24^ both on‐ and off‐label, among other medications, are frequently used to manage aggression and impulsivity in these disorders. However, these drugs exhibit significant side effects, as discussed above. Benzodiazepines, mood stabilizers, and stimulants such as methylphenidate, although less frequently used, are also prescribed for patients with ADHD, but their efficacy in controlling aggression is only mild in most of the cases and are not free of side effects such as digestive disturbances, dizziness, anxiety, or increased heart rate, which, in some cases, can lead to cardiovascular issues.^19^, ^25^

REIMAGINE^26^ was designed as a basket study using a methodology similar to that of oncology basket trials, to test the effect of vafidemstat as a nonsedative treatment for aggression in BPD, ADHD, and ASD. The study employed measures of A/A, as well as clinical outcome assessments that measured the effects on overall patient functioning.

## Methods

### Study design and participant selection

The REIMAGINE trial was a phase IIa, single‐center, open‐label, single‐arm study. It consisted of a screening period (up to 1 week) that was followed by an 8‐week treatment period and a 4‐week follow‐up period. Seven weekly/biweekly visits—baseline (visit 1) to end of study (visit 7)—were completed during the treatment period, and two safety visits at 14 and 28 days after the last dose were completed during the 4‐week posttreatment follow‐up period. The total duration of the study was 13 weeks. Participants who discontinued the study had to complete the assessments planned for the end of study visit as soon as possible and were asked to attend the 4‐week safety follow‐up visit (Fig. S1).

A total of 32 participants (from 35 screened participants) were included in the study between October 2018 and August 2019. Participants were enrolled from a single center in Spain (Hospital Universitari Vall d'Hebron‐Department of Psychiatry) and allocated as follows: 12 ADHD, 13 BPD, and seven ASD. The original protocol included two additional populations (Alzheimer disease and Lewy Body Dementia) but these were excluded after the start of the trial because of lack of eligible participants at the investigational site. Informed consent to participate in the study was obtained from all participants after the study and procedures had been fully explained. No formal sample size estimation was conducted.

The REIMAGINE protocol was designed as a basket trial to investigate A/A with presumed similar underlying mechanistic cause across different CNS conditions: ADHD, BPD, and ASD. To enter the trial, participants were required to exhibit significant or persistent A/A that was disruptive to their daily living or put them in harm's way for at least 3 days per week for at least 4 weeks prior to the screening visit. Patients entering the study under any pharmacological treatment for ADHD, BPD, or ASD had to be on a stable dose for at least 1 month prior to the screening visit and had to remain on the same dose throughout the study. The main inclusion and exclusion criteria are summarized in Table S1.

Vafidemstat was administered orally at 1.2 mg/day in a single capsule under fasting conditions, once daily in a 5‐days‐on/2‐days‐off schedule for 8 weeks.

Details on the planned assessments are displayed in Figure S2.

Figure S3 illustrates the flow of participants throughout the study.

The primary objective of REIMAGINE was to evaluate safety and tolerability; the secondary objective was to investigate the efficacy of treating A/A in adult patients with ADHD, ASD, and BPD; exploratory objectives comprised pharmacokinetic (PK) and pharmacodynamic (PD) evaluations. The study was approved by the ethics committee of the Hospital Universitari Vall d'Hebron and performed in accordance with the current version of the Declaration of Helsinki (52nd WMA General Assembly, Edinburgh, Scotland, October 2000) as adopted by the World Medical Association, and was conducted in agreement with the ICH guidelines on Good Clinical Practice.

### Primary end points: safety and tolerability assessments

Safety and tolerability assessments included the following: (i) physical examination, i.e. height (at screening only), body weight, and body mass index; (ii) vital signs (supine diastolic and systolic blood pressure, supine heart rate, supine respiration rate, resting respiration rate, digital axillary temperature); (iii) electrocardiography (ECG); (iv) treatment‐emergent adverse events (TEAEs) and serious TEAEs; (v) clinical laboratory analyses (hematology and biochemistry); and (vi) use of concomitant medication.

The causal relationship of an adverse event (AE) with the study medication was established according to the World Health Organization Causality Assessment.^27^ All AEs judged as having a reasonable suspected causal relationship to the treatment (i.e. certainly, probably, possibly) were considered as related to treatment.

As part of the safety assessment, all participants completed the Columbia‐Suicide Severity Rating Scale (C‐SSRS), an interview developed to systematically assess suicidal ideation and its intensity (five questions) and suicidal behavior (four questions).^28^, ^29^

### Secondary end points: efficacy assessments

The efficacy of vafidemstat in treating A/A was evaluated by the Clinical Global Impression for Severity (CGI‐S) and Clinical Global Impression for Improvement (CGI‐I), and by the four‐item Neuropsychiatric Inventory (NPI) questionnaire for Agitation‐Aggression (NPI‐A/A) scale. The overall patient functioning was evaluated using the total NPI scale and the following disease‐specific scales: Attention‐Deficit/Hyperactivity Disorder Rating Scale (ADHD‐RS) for patients with ADHD and Borderline Personality Disorder Checklist (BPDCL) for patients with BPD. The Autism Diagnostic Observation Schedule (ADOS) was used for patients with ASD only for diagnostic purposes and not for efficacy evaluation.

#### CGI‐S and CGI‐I

Illness severity (CGI‐S) and global change or improvement (CGI‐I) developed by Webster (1976) assessed the severity and the improvement (or worsening) of A/A behavior on a seven‐point scale.

#### Total NPI and NPI‐A/A

The 12‐item NPI questionnaire as developed by Cummings^30^ (three‐point severity scores) was used to assess overall patient functioning based on the following 12 items: delusions, hallucinations, A/A, dysphoria/depression, anxiety, euphoria, apathy, disinhibition, irritability, aberrant motor disturbance, nighttime behavioral disturbances, and appetite and eating abnormalities. Total NPI score was calculated by adding all the item scores. The NPI‐A/A subscale score was calculated by adding only the individual scores of the four items related to A/A (agitation/aggression, disinhibition, irritability, and aberrant motor disturbance).

#### Disease‐specific scales

##### ADHD Rating Scale

The ADHD‐RS is an 18‐item scale, self‐report version, for assessing symptoms for ADHD DSM‐IV, also consistent with those of the DSM‐5. The Spanish version of the ADHD‐RS was validated in 2017 and discriminates between adults with ADHD and controls.^31^

##### Borderline Personality Disorder Checklist

The BPDCL is a DSM‐IV–based PRO designed to assess the extent to which respondents have been bothered by a range of BPD symptoms, over the course of the past month. Responses to the 47 items range from “not at All” (1) to “extremely” (5). Validation studies have demonstrated that the BPDCL discriminates between individuals with and without a diagnosis of BPD, and that it is sensitive to change of BPD symptoms.^32^, ^33^ Overall, the BPDCL has good to excellent internal consistency, as well as very good discriminant, convergent, and construct validity. Finally, as the confirmatory factor analysis on the BPDCL supported a nine‐factor model aligned with the DSM‐IV (as well as now DSM‐V) BPD diagnostic criteria, construct validity has been firmly established.

### Exploratory end points

PK and PD assessments included monitoring of vafidemstat trough concentrations in plasma and KDM1A target engagement (TE) in peripheral blood mononuclear cells (PBMCs), respectively, during the first month of treatment.

#### Pharmacokinetics

Vafidemstat concentrations were determined in human plasma by using a highly sensitive validated LC–MS/MS method (lower limit of quantification: 10 pg/mL) with electrospray ionization in positive ion mode and deuterated vafidemstat as internal standard, developed at Laboratorios Echevarne, S.A, Spain.

#### Pharmacodynamics

KDM1A TE (percentage of KDM1A bound to vafidemstat) was determined in PBMCs by using a proprietary chemoprobe‐based assay developed at Oryzon Genomics S.A., Spain.^34^, ^35^

### Data management and analysis

Data management and handling of data was conducted according to the study‐specific data management plan, which followed the International Conference on Harmonization (ICH) guidelines at the Vall d'Hebron Research Institute (VHIR). Written and electronic case report form systems were used to collect data, and validation and data queries were handled by VHIR.

The safety analysis set (SAF) population, i.e. all participants who received at least one dose of vafidemstat (*n* = 32), was used for safety and tolerability analysis. The full analysis set (FAS) population, i.e. all participants who received at least one dose of vafidemstat and completed at least one clinical assessment (*n* = 32), was used for PK/PD analyses. The primary population for efficacy evaluation was the per‐protocol set (PPS), i.e. those participants who completed the assessment of at least one primary end point at visit 7 (week 8) with no major protocol violations (*n* = 23).

Change from visit 1 (baseline) to any other visits was assessed: (i) for each participant; (ii) for all participants (aggregated data); and (iii) for each different cohort (i.e. ADHD, ASD, and BPD). A sensitivity analysis was additionally conducted in the FAS population by considering the PPS last‐observation‐carried‐forward (LOCF), which allowed imputing missing data. Statistical comparisons between measurements were performed using nonparametric tests, such as the Wilcoxon signed rank test, the Friedman test, or the one‐tailed paired *t* test, depending on the nature and distribution of the variables. Dunn multiple comparison test was used as post hoc test when applicable. Correlations were assessed by means of Pearson coefficient. Data analyzed with Wilcoxon signed rank or the Friedman tests were additionally analyzed using the corresponding parametric tests, with comparable results (not shown). All statistical tests were performed at a level of statistical significance of 0.05.

## Results

### Patient characteristics

The SAF population consisted of 18 (56%) female and 14 (44%) male participants, and the majority (84%) were of White race. The median age was 30 years (range between 19 and 64 years). Most participants were school graduates (53%) or had a college/university degree (28%). No significant differences in baseline physical conditions and vital signs were observed, except for body weight and, consequently, body mass index, which were higher in the ASD group (Table 1).

**Table 1:** Summary of baseline patient characteristics

		All (*N* = 32)	ADHD (*n* = 12)	BPD (*n* = 13)	ASD (*n* = 7)
Age (years)		30 (19–64)	28.5 (19–64)	34 (19–46)	30 (20–44)
Sex	Female	18 (56%)	4 (33%)	13 (100%)	1 (14%)
	Male	14 (44%)	8 (67%)	0 (0%)	6 (86%)
Race/ethnicity	White	27 (84%)	9 (75%)	12 (92%)	6 (86%)
	Latin	5 (16%)	3 (25%)	1 (8%)	1 (14%)
Education	Some school	6 (19%)	3 (25%)	2 (15%)	1 (14%)
	School graduate	17 (53%)	7 (58%)	6 (46%)	4 (57%)
	College graduate	8 (25%)	2 (17%)	4 (31%)	2 (29%)
	University degree	1 (3%)	0 (0%)	1 (8%)	0 (0%)
Height (m)		1.70 (1.52–1.93)	1.71 (1.52–1.93)	1.63 (1.53–1.72)	1.78 (1.75–1.90)
Body weight (kg)		70.0 (47.7–150)	68.7 (51.4–101)	59.2 (47.7–94.5)	105 (68.5/150)
Body mass index (kg/m^2^)		24.37 (18.59–48.98)	23.64 (18.98–35.79)	21.49 (18.59–34.09)	30.68 (22.5/48.98)

Data are presented as median (range) for age, height, body weight, and body mass index. For the rest of variables, data represent the number of participants (percentage of total participants of the cohort).

ADHD, attention‐deficit/hyperactivity disorder; ASD, autistic spectrum disorder; BPD, borderline personality disorder.

Psychiatric disorders, including the three study conditions (ADHD, BPD, and ASD) as well as substance use, general anxiety, and eating disorders, made up the majority of reported medical histories (82.1% reported by 100% of the participants) (Table S2). A total of 29 participants (90.6%) reported concomitant medications during the treatment period, with psychoanaleptics being the most commonly used (20.3%, by 50% of the participants) (Table S3).

A total of nine participants (four with BPD, four with ADHD, one with ASD) did not complete the study treatment. Reasons for treatment discontinuation were withdrawal of informed consent (five), lost to follow‐up (three), or unmet eligibility criteria (one) (Fig. S3).

### Safety and tolerability

Twenty‐four of 32 (75%) participants reported a total of 96 AEs. Of those, 43 AEs (reported in 15 participants) were considered related to vafidemstat treatment (six certainly, 19 probably, 17 possibly, and one not being assessable) according to investigator criteria (Table 2) and thus defined as adverse reactions (ARs). All ARs were mild events that resolved without intervention. The most common AR was headache (*n* = 18, in four participants), followed by overdose (*n* = 6, in two participants), anxiety (*n* = 4, in three participants), abnormal behavior (*n* = 2, in one patient), discomfort (*n* = 2, in one patient), and constipation (*n* = 2, in two participants). All other ARs were thrombocytopenia, abdominal pain, diarrhea, dry mouth, fatigue, oral herpes, sensory disturbance, epistaxis, and eczema, each reported only once in one patient. There were no serious AEs and none of the participants discontinued the treatment for safety reasons.

**Table 2:** AEs classified as related to the study drug (ARs)

System organ class Preferred term	Number of patients (%)	Event count
Nervous system disorders	5 (15.6)	19
Headache	4 (12.5)	18
Sensory disturbance	1 (3.1)	1
Gastrointestinal disorders	5 (15.6)	5
Constipation	2 (6.3)	2
Abdominal pain	1 (3.1)	1
Diarrhea	1 (3.1)	1
Dry mouth	1 (3.1)	1
Psychiatric disorders	4 (12.5)	6
Anxiety	3 (9.4)	4
Abnormal behavior	1 (3.1)	2
Injury, poisoning, and procedural complications	2 (6.3)	6
Overdose	2 (6.3)	6
General disorders and administration site conditions	2 (6.3)	3
Discomfort	1 (3.1)	2
Fatigue	1 (3.1)	1
Blood and lymphatic system disorders	1 (3.1)	1
Thrombocytopenia	1 (3.1)	1
Infections and infestations	1 (3.1)	1
Oral herpes	1 (3.1)	1
Respiratory, thoracic, and mediastinal disorders	1 (3.1)	1
Epistaxis	1 (3.1)	1
Skin and subcutaneous tissue disorders	1 (3.1)	1
Eczema*	1 (3.1)	1

Percentages are based on total number of patients (*N* = 32). A patient with more than one finding in a specific system organ class or preferred term category was only counted once.

* Unassessable. AE, adverse event; AR, adverse reaction.

There were only six laboratory results outside the normal range in the physical examination, vital signs, ECG, and hematology/biochemistry evaluations. None of them were deemed clinically significant and only one, a mild thrombocytopenia that spontaneously resolved, was associated to vafidemstat treatment.

**Fig. 1:**
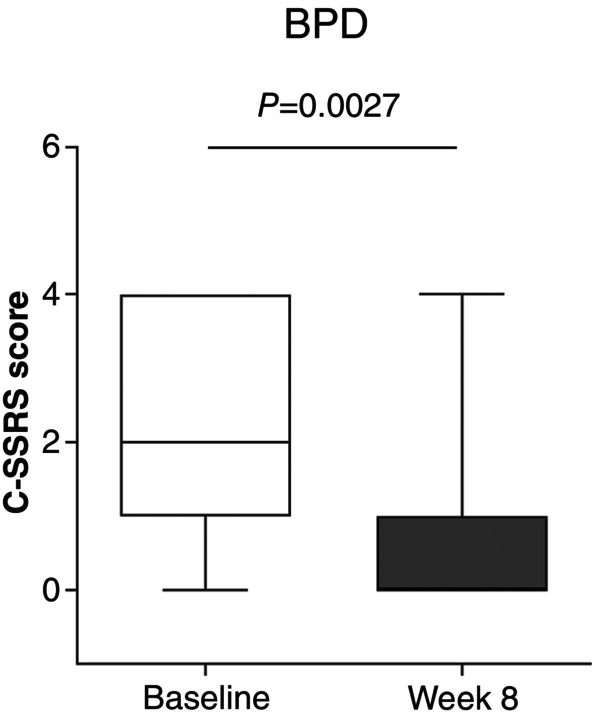
Effect of vafidemstat on suicidal ideation/behavior in patients with borderline personality disorder (BPD). Effect of vafidemstat on suicidal ideation/behavior in participants with BPD assessed by the Columbia‐Suicide Severity Rating Scale (C‐SSRS). Results are presented as Tukey whisker plots with outliers. Wilcoxon signed rank test was used for statistical comparisons.

According to the C‐SSRS scores, the risk of suicidal ideation and/or suicidal behavior did not increase in the total study population, supporting the safe use of vafidemstat in psychiatric disorders. Notably, a significant reduction of suicidal ideation was observed in participants with BPD, the only study cohort where this trait is relevant (Fig. 1). In the other two cohorts, only two patients with ASD and no patients with ADHD reported one episode of suicidal ideation in the preceding 12 months of study participation, and none during the trial (data not shown).

Based on the above, vafidemstat was safe and well tolerated and met the primary end point of the trial. Safety data were comparable to all previous vafidemstat clinical studies to date.

### Efficacy

#### Aggression

**Fig. 2:**
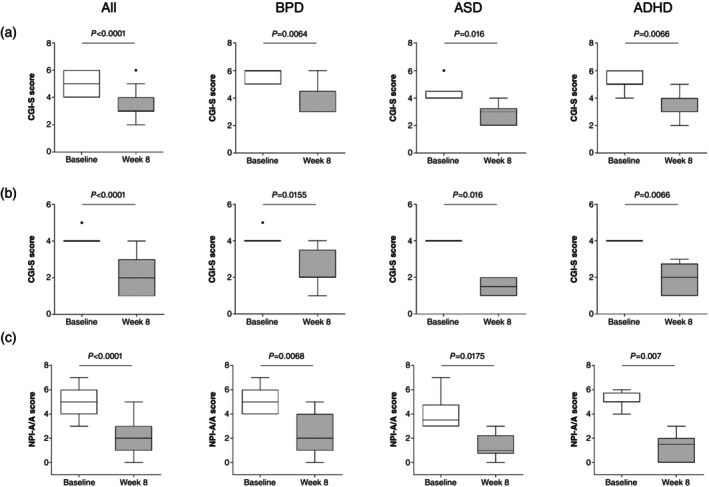
Vafidemstat efficacy on aggression. End of treatment. Effect of vafidemstat on aggression was assessed by the Clinical Global Impression for Severity (CGI‐S) (a), Clinical Global Impression for Improvement (CGI‐I) (b), and Neuropsychiatric Inventory questionnaire for Agitation‐Aggression (NPI‐A/A) (c) scales in all participants. Baseline and end‐of‐treatment (week 8) data are presented as Tukey whisker plots with dots representing outliers. Wilcoxon signed rank test was used for statistical comparisons.

**Fig. 3:**
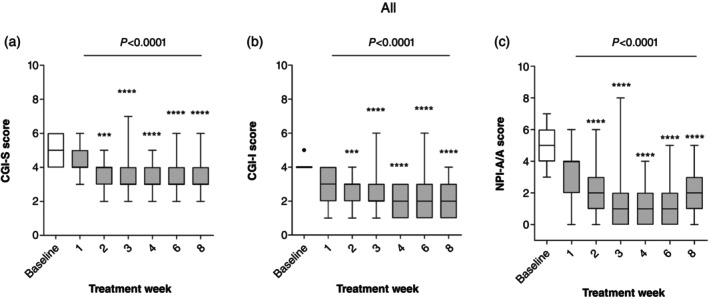
Vafidemstat efficacy on aggression. Change over time. Effect of vafidemstat on aggression was assessed by the Clinical Global Impression for Severity (CGI‐S) (a), Clinical Global Impression for Improvement (CGI‐I) (b), and Neuropsychiatric Inventory questionnaire for Agitation‐Aggression (NPI‐A/A) (c) scales in all participants. Change over time in the respective scores is presented as Tukey whisker plots with dots representing outliers. Friedman and Dunn multiple comparison post hoc tests were used for statistical comparisons (***P < 0.001, ****P < 0.0001).

Eight‐week vafidemstat treatment led to a statistically significant reduction in A/A compared with baseline across all the assessments, i.e. CGI‐S (Fig. 2a), CGI‐I (Fig. 2b), and NPI‐A/A (Fig. 2c), both in the analyses of the aggregated data (all participants, *P* < 0.0001) and of each of the three disease groups (*P*‐values ranging from 0.0064 to 0.0175). Importantly, these changes occurred as early as within the first 2 weeks of treatment and were sustained throughout the treatment period (*P* < 0.0001) (Fig. 3a–c). These results obtained for the PPS population were further confirmed in the sensitivity analysis (LOCF) performed on the FAS population (data not shown), thus suggesting that patient withdrawal did not affect the efficacy readouts.

#### Overall patient functioning

**Fig. 4:**
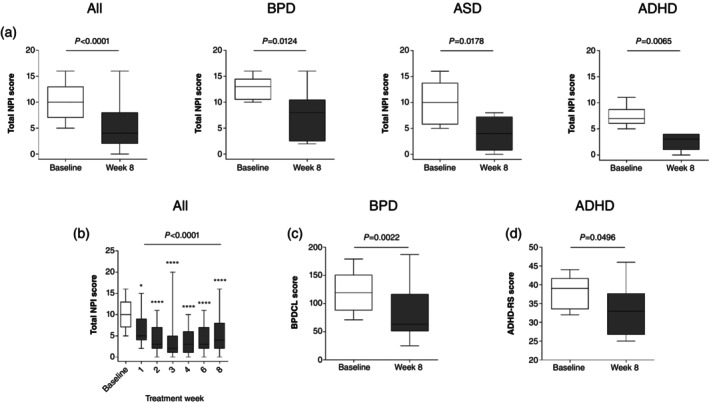
Efficacy of vafidemstat on overall patient functioning. Effect of vafidemstat on overall patient functioning, including total Neuropsychiatric Inventory (NPI) scores (a,b) for all participants and disease‐specific scales for participants with borderline personality disorder (BPD) assessed by the Borderline Personality Disorder Checklist (BPDCL) (c) and participants with attention‐deficit/hyperactivity disorder (ADHD) assessed by the Attention‐Deficit/Hyperactivity Disorder Rating Scale (ADHD‐RS) (d). Results are presented as Tukey whisker plots with dots representing outliers. Wilcoxon signed rank (a), Friedman and Dunn multiple comparison post hoc (b), or paired *t* test (c,d) were used for statistical comparisons (****P < 0.0001).

Eight‐week vafidemstat treatment resulted in a significant improvement in overall patient functioning compared with baseline, as assessed by the total NPI scores (Fig. 4a), both for the aggregated data (*P* < 0.0001) and each of the three disease groups (*P*‐values 0.0065–0.0178). Changes were already evidenced from the first weeks of treatment (*P* < 0.0001) (Fig. 4b).

Assessment of BPD and ADHD disease‐specific scales further confirmed improved patient functioning. In particular, a statistically significant reduction in the BPDCL total score was observed after 8 weeks of treatment with vafidemstat compared with baseline (*P* = 0.0022) (Fig. 4c). Noteworthy, scores in three participants went down to levels below the diagnostic threshold for BPD according to the BPDCL that aligns with DSM‐5 criteria for this disorder. After 8 weeks of treatment, a statistically significant decrease was also observed in the ADHD‐RS score compared with baseline (*P* = 0.0496) (Fig. 4d).

**Fig. 5:**
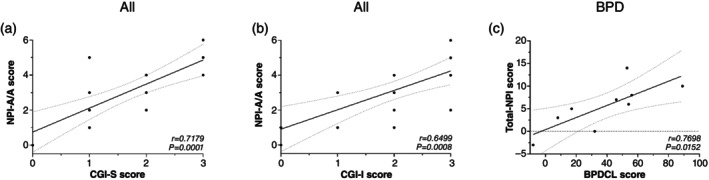
Correlation between clinical scores. Pearson correlation coefficient (*r*) and *P*‐value between aggression scores: Clinical Global Impression for Severity (CGI‐S) and Neuropsychiatric Inventory questionnaire for Agitation‐Aggression (NPI‐A/A) (a) and Clinical Global Impression for Improvement (CGI‐I) and NPI‐A/A (b) for all participants, and between overall functioning scores for participants with borderline personality disorder (BPD): Borderline Personality Disorder Checklist (BPDCL) and total Neuropsychiatric Inventory (NPI) (c).

Remarkably, statistically significant correlations were observed between the clinical scores assessing aggression (NPI‐A/A vs CGI‐S and CGI‐I) (Fig. 5a,b), and between those evaluating overall patient functioning (total NPI vs BPDCL) (Fig. 5c). This convergence of signals in scales of different nature and scope supports the pharmacological role of vafidemstat in controlling A/A in different psychiatric conditions.

### Pharmacokinetics

**Fig. 6:**
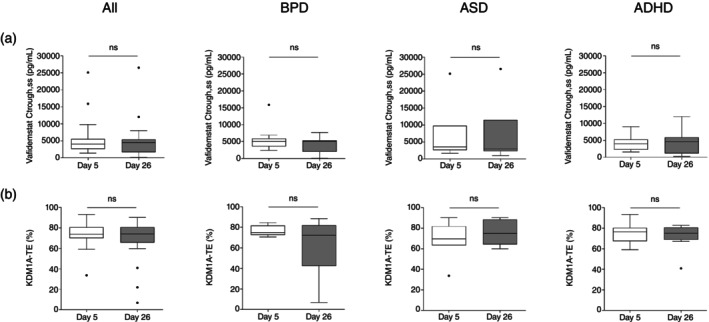
Pharmacokinetics and KDM1A target engagement (TE) of vafidemstat. Plasma levels of vafidemstat (upper panels) and KDM1A TE in peripheral blood mononuclear cells (bottom panels) at pre‐dose (<lower limit of quantification or 0, not shown) and trough samples on day 5 and 26 of treatment. Results are presented as Tukey whisker plots for all participants (left panels) and for each of the three disease groups independently (borderline personality disorder [BPD], autistic spectrum disorder [ASD], and attention‐deficit/hyperactivity disorder [ADHD] panels). The dots in the panels represent outliers. Wilcoxon signed rank test was used for statistical comparisons. ns, not significant.

There were no statistically significant differences in vafidemstat plasma trough concentrations (Ctrough) between day 5 and 26, suggesting that steady‐state concentrations were reached within the first week of treatment. Drug exposure in terms of Ctrough was comparable in all three disease cohorts (Fig. 6a).

### KDM1A target engagement

There were no statistically significant differences between the PBMC KDM1A TE values observed on day 5 and 26, with most of the participants reaching a maximum effect of ≈70% to 80% TE within the first week of treatment. No statistically significant differences were observed among the KDM1A TE values of each of the different disease groups (Fig. 6b).

## Discussion

KDM1A in humans plays an important role in CNS development in the control of proliferation of neural stem cells and their differentiation.^36^ In mice, its importance for neuronal differentiation, migration, and morphology during corticogenesis has also been established.^37^ After birth, KDM1A partly changes its function, with neuronal KDM1A isoforms coordinating early neurite morphogenesis and dynamic maintenance of the neuronal phenotype through the regulation of different target genes.^38^

We previously showed that, by targeting KDM1A, vafidemstat improves memory, reduces aggression, and increases sociability in rodents and modulates the transcription of IEGs in the prefrontal cortex in response to stress.^8^ Based on these findings, REIMAGINE was conducted to assess whether treatment with vafidemstat would be effective to reduce A/A across overlapping but distinct CNS disorders in humans.

REIMAGINE was designed as an open‐label exploratory basket trial to gain initial proof of concept, which would inform the design of further controlled clinical trials. The hypothesis was that the transcriptional effects of vafidemstat may also be a downstream mechanism and hence produce the same response to A/A in several distinct psychiatric disorders such as BPD, ADHD, and ASD, which might be assessed by validated clinical scales.

The study has provided several positive outcomes. First, it is one of the first basket studies performed in CNS and allowed confirmation of A/A as a common feature that could serve as a therapeutic target across several psychiatric disorders. Second, vafidemstat was proven to be safe and well tolerated in all the tested cohorts, thereby meeting the primary end point of the trial. In addition, it showed significant efficacy signals in a population sample that, based on baseline scale scores, had serious illness. Third, it constituted the first evidence that vafidemstat plasma exposure and KDM1A TE in polymedicated participants from distinct pathologies are in accordance with those observed earlier in healthy volunteers.

This basket trial also represents a novel attempt at bringing precision medicine to psychiatry. Abnormalities in the methylation‐demethylation balance and mutations or deletions in genes involved in synaptic transmission have been characterized in several psychiatric and neurological conditions.^39^, ^40^, ^41^ A number of preclinical studies have mechanistically linked KDM1A to multiple genetically defined CNS disorders. These include KDM1A inhibition rescuing neuronal branching deficits, and circuitry and cognitive deficits in the *Setd1a*‐deficiency schizophrenia (SCZ) mouse model,^7^ hippocampal neurogenesis impairment and memory defects in the KMT2D Kabuki syndrome model,^42^ impaired learning functions in the N‐methyl‐D‐aspartate (NMDA) receptor hypofunction SCZ mouse model,^43^ and autism‐like behavioral abnormalities and restoring NMDA receptor function in the prefrontal cortex and AMPA receptor‐mediated currents in striatum of Shank3‐deficient mice in the *Shank3* Phelan‐McDermid (a variant of ASD) mouse model.^44^ These distinct rescues argue for a downstream role of KDM1A, which might be acting as a hub where several defective pathways would converge and, hence, may also constitute the basis for future basket trials based on those molecular deficiencies. The phenotypic rescues in NMDAR‐SCZ and in the *Shank3*‐ASD mice models reports suggestively converge with published data that propose that multiple cognitive dysfunctions and symptoms presented by patients with BPD, such as dissociation, psychosis, and impaired nociception, may result from the dysregulation of the NMDA neurotransmission.^45^

The positive safety and efficacy results obtained in REIMAGINE offer face validity and are useful for the design of future controlled clinical studies with vafidemstat in psychiatric disorders. The REIMAGINE safety data are aligned with the results from previous or coetaneous phase IIa studies, which include more than 400 individuals exposed to vafidemstat for periods of between 1 day to more than 2 years. This large safety data set suggests that vafidemstat is well tolerated and could represent an important benefit over current therapeutic alternatives, which are typically associated with numerous side effects, including extrapyramidal symptoms, sedation, sexual anhedonia, or weight gain,^22^ none of which are detected in patients treated with vafidemstat.

This clinical trial has limitations such as its open‐label design, performed in a single center and with a small number of participants. Yet, with all the necessary cautions, the magnitude and consistency of treatment effects suggest that vafidemstat can offer therapeutic benefits to treat A/A across distinct psychiatric conditions. Of note, the significant reduction in A/A and improvement in overall functioning occurred within 1 to 2 weeks of treatment and remained stable throughout the study, thus, suggesting that such benefits can be rapid and sustained long term. The consistency argues that there is a common underlying mechanistic reason for this reduction in A/A. This approach is grounded in the original scientific prediction of a shared downstream mode of action, rather than merely suppressing symptoms as antipsychotics do. It may involve restoring the balance of immediate early gene expression in the prefrontal cortex, as observed in preclinical models. However, further research is needed to determine whether this is the primary mechanism or one among others at play.

Further encouragement comes from the reduction in the nonaggression features of these psychiatric diseases and the improvement seen in the overall scales for the individual conditions, which support vafidemstat as having further therapeutic effects beyond the treatment of A/A. This is important, as these are conditions that still have a high unmet medical need, both in terms of detection and pharmacological options (which are either absent or associated with severe side effects). It is therefore expected that a new and effective therapeutic option, such as vafidemstat, with a good safety and tolerability profile could increase adherence in long‐term therapy.

These efficacy signals, together with preclinical and genetic evidence, have been the basis for subsequent clinical trials with vafidemstat in psychiatric disorders. The first of these is PORTICO, an adaptive double‐blind placebo‐controlled phase IIb trial in patients with BPD,^46^, ^47^ with multiple primary end points, including overall BPD improvement and A/A reduction. Moreover, the role of KDM1A in schizophrenia is the rationale for EVOLUTION, an adaptive double‐blind placebo‐controlled phase IIb trial in schizophrenia,^48^ with multiple end points including improvement in negative symptoms and in cognitive impairment associated with schizophrenia.

In conclusion, REIMAGINE was a novel experimental study, with a design borrowed from oncology, to explore whether a single therapeutic agent, vafidemstat, has a mode of action that is effective in the control of aggression in multiple psychiatric indications. The study showed that vafidemstat is safe and well‐tolerated and elicits significant and consistent reduction in A/A in patients with BPD, ADHD, and ASD. Vafidemstat also produced a significant improvement in nonaggressive features and overall disease indicators in these populations. These data support the continued development of vafidemstat as a new therapeutic option in multiple psychiatric disorders.

## Disclosure statement

MF and LG report fees from Vall d'Hebron University Hospital. In addition, LG received fundings from Shire to attend symposia. VR was on the speakers' bureau and/or acted as consultant for Takeda, Eli‐Lilly. She also received travel awards for taking part in psychiatric meetings from Janssen‐Cilag, Rubió, Takeda, and Eli‐Lilly. JX, SG, MIA, MR, RB, and CB were Oryzon Genomics employees during the conduct of the study. CB is executive director and shareholder of Oryzon Genomics. JARQ was on the speakers' bureau and/or acted as consultant for Eli‐Lilly, Janssen‐Cilag, Novartis, Shire, Takeda, Bial, Shionogui, Lundbeck, Almirall, Braingaze, Sincrolab, Medice and Rubió, Raffo. He also received travel awards for taking part in psychiatric meetings from Janssen‐Cilag, Rubió, Shire, Takeda, Shionogui, Bial, Medice and Eli‐Lilly. The Department of Psychiatry chaired by him received unrestricted educational and research support from the following companies: Eli‐Lilly, Lundbeck, Janssen‐ Cilag, Actelion, Shire, Ferrer, Oryzon, Roche, Psious, and Rubió. CB, RB and JARQ are listed as inventors in some of Oryzon's patents.

## Author contributions

M.F., V.R., J.X., S.G., M.R., R.B., C.B., and J.A.R.Q.—conceptualization and design of the study. M.F., V.R., L.G., J.X., M.I.A., and J.A.R.Q.—acquisition and analysis of data. M.F., J.X., S.G., M.R., R.B., C.B., and J.A.R.Q.—supervision, project administration. All writing‐review & editing and final approval.

## Supporting information


**Figure S1.** Study design.

**Figure S2.** Flowchart of planned assessments.

**Figure S3.** Consort diagram.

**Table S1.** Main eligibility criteria.
**Table S2.** Medical history.
**Table S3.** Concomitant medications.
