# Supplementary figures and images for: REIMAGINE: A central nervous system basket trial showing safety and efficacy of vafidemstat on aggression in different psychiatric disorders
Source: Psychiatry Clin Neurosci. 2025 Feb 12;79(5):257–65. doi: 10.1111/pcn.13800 (PMC12047063; doi:10.1111/pcn.13800)

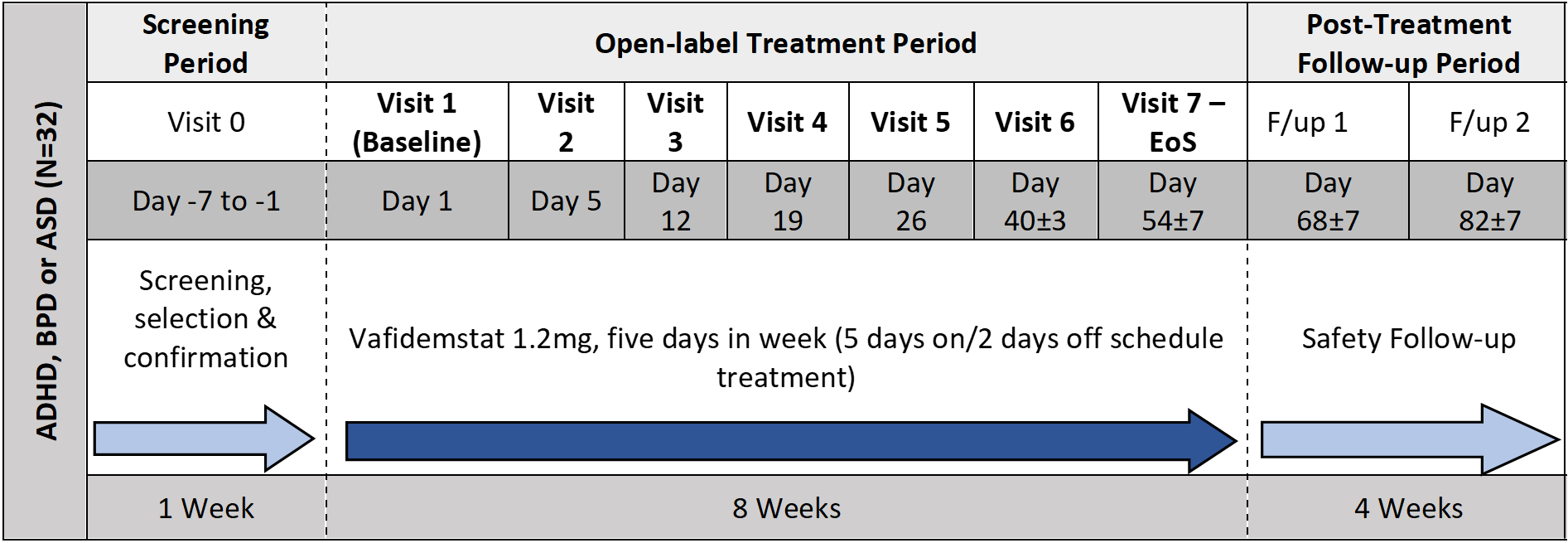

Supplement: Supplementary file 1 — Figure S1. Study design. [file PCN-79-257-s003.tif]

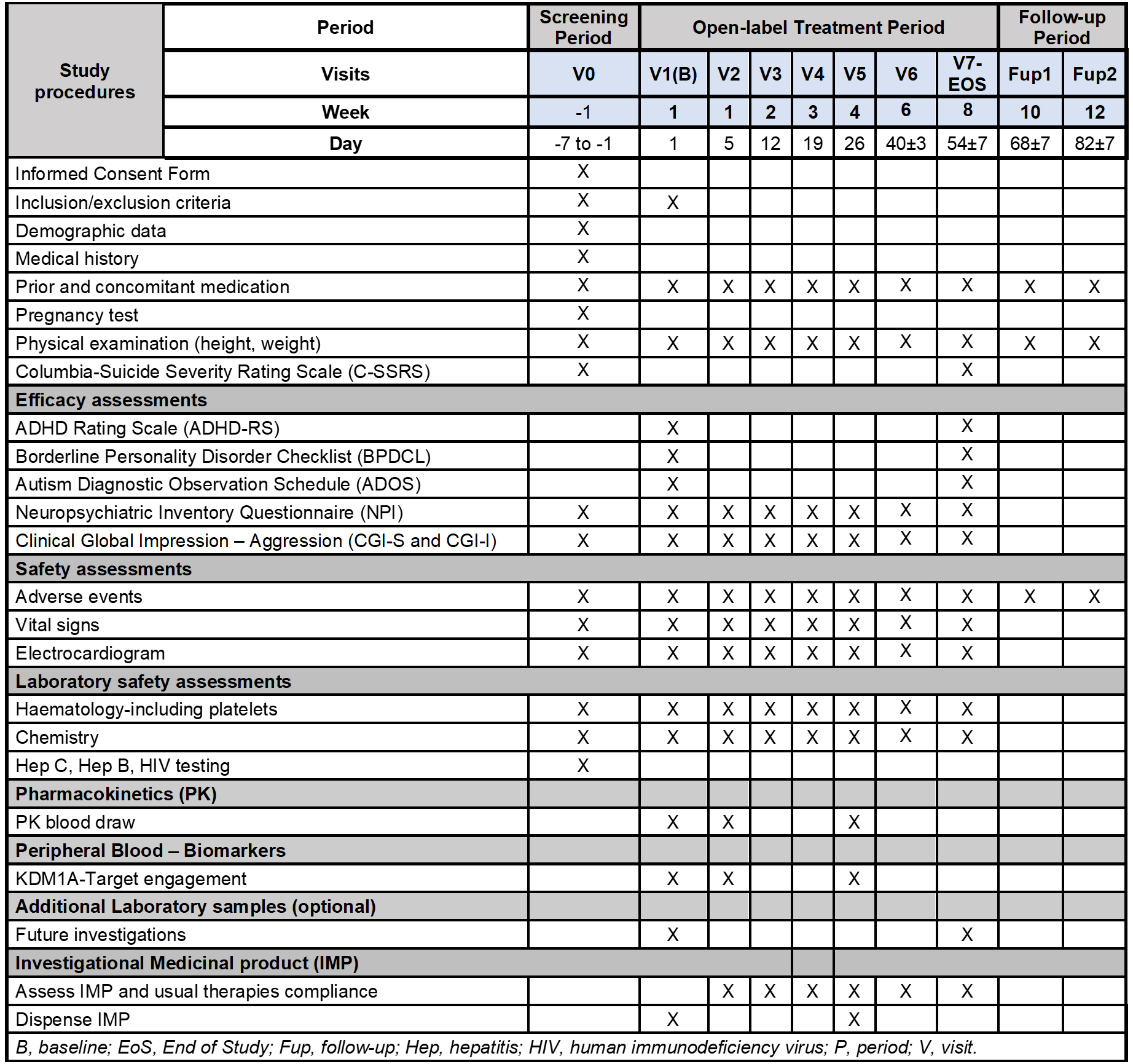

Supplement: Supplementary file 2 — Figure S2. Flowchart of planned assessments. [file PCN-79-257-s002.tif]

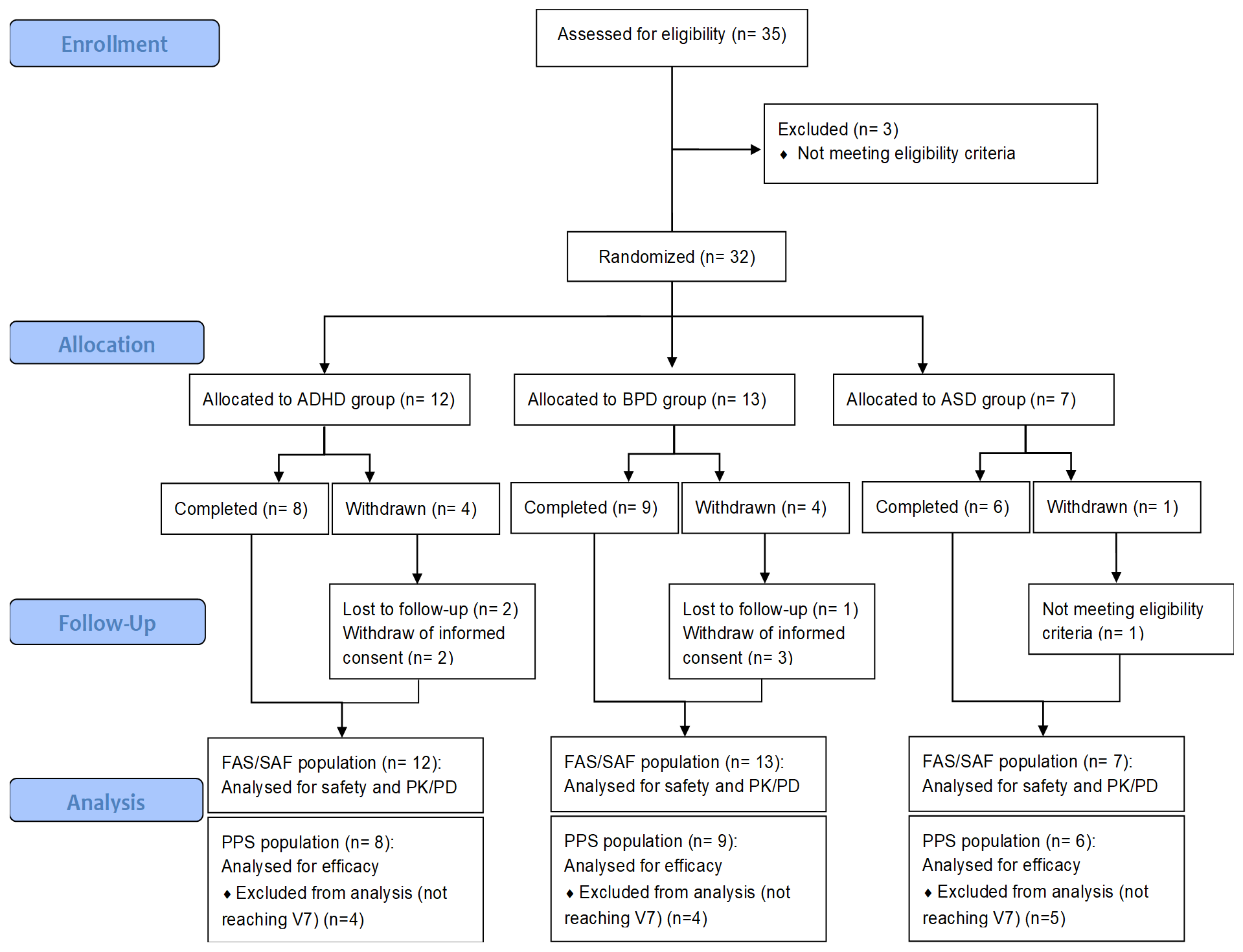

Supplement: Supplementary file 3 — Figure S3. Consort diagram. [file PCN-79-257-s004.tif]
