# Supplementary material for: REIMAGINE: A central nervous system basket trial showing safety and efficacy of vafidemstat on aggression in different psychiatric disorders
Source: Psychiatry Clin Neurosci. 2025 Feb 12;79(5):257–65. doi: 10.1111/pcn.13800 (PMC12047063; doi:10.1111/pcn.13800)
Supplement: Supplementary file 4 — Table S1. Main eligibility criteria. Table S2. Medical history. Table S3. Concomitant medications. [file PCN-79-257-s001.docx]

# **Supplemental Tables**

Supp Table 1 Main eligibility criteria.

| **Inclusion Criteria** | **Exclusion Criteria** |
| --- | --- |
| - Men and women 18-85 years of age. | - Failure to perform screening or baseline examinations. |
| - Significant or persistent agitation or aggression that was disruptive to patient’s daily living or put the patient in harm´s way for at least 3 days per week for at least 4-weeks prior to screening visit. | - Hospitalization or change of concomitant medication 1 month prior to Screening visit or during Screening Period (partial hospitalization is considered outpatient). |
| - Outpatient | - Clinically significant, advanced or unstable disease that may interfere with evaluation (those with clinically significant indicators of instability) |
| - A current diagnosis for ADHD, BPD or ASD according to DSM-5 criteria. | - Disability that may prevent the patients from completing all study requirements; for instance, blindness, deafness, severe language difficulty. |
| - If prescribed, stable pharmacological treatment (i.e.: with the same dose) as per SmPC of ADHD, BPD or ASD for at least one month prior to screening, including anti-inflammatories. | - Chronic drug intake of forbidden medication as specified in the clinical stud protocol |
| - Fertile male and female subjects must use highly efficient contraception. | - Suspected or known drug or alcohol abuse. |
| - Signed informed consent | - Suicide attempt within the last year or significant risk of suicide (in the opinion of the investigator, defined as a “yes” to suicidal ideation questions 4 or 5, or answering “yes” to suicidal behaviour on the Columbia-Suicide Severity Rating Scale within the past 12 months). |
|  | - Any condition that in the opinion of the investigator makes the patient unsuitable for inclusion in the study. |

Supp Table 2 Medical history.

Supp Table 3 Concomitant medications.
